# Supplementary material for: Evolution of a ZW sex chromosome system in willows
Source: Nat Commun. 2023 Nov 6;14:7144. doi: 10.1038/s41467-023-42880-5 (PMC10628195; doi:10.1038/s41467-023-42880-5)
Supplement: Supplementary file 1 — Supplementary information [file 41467_2023_42880_MOESM1_ESM.pdf]

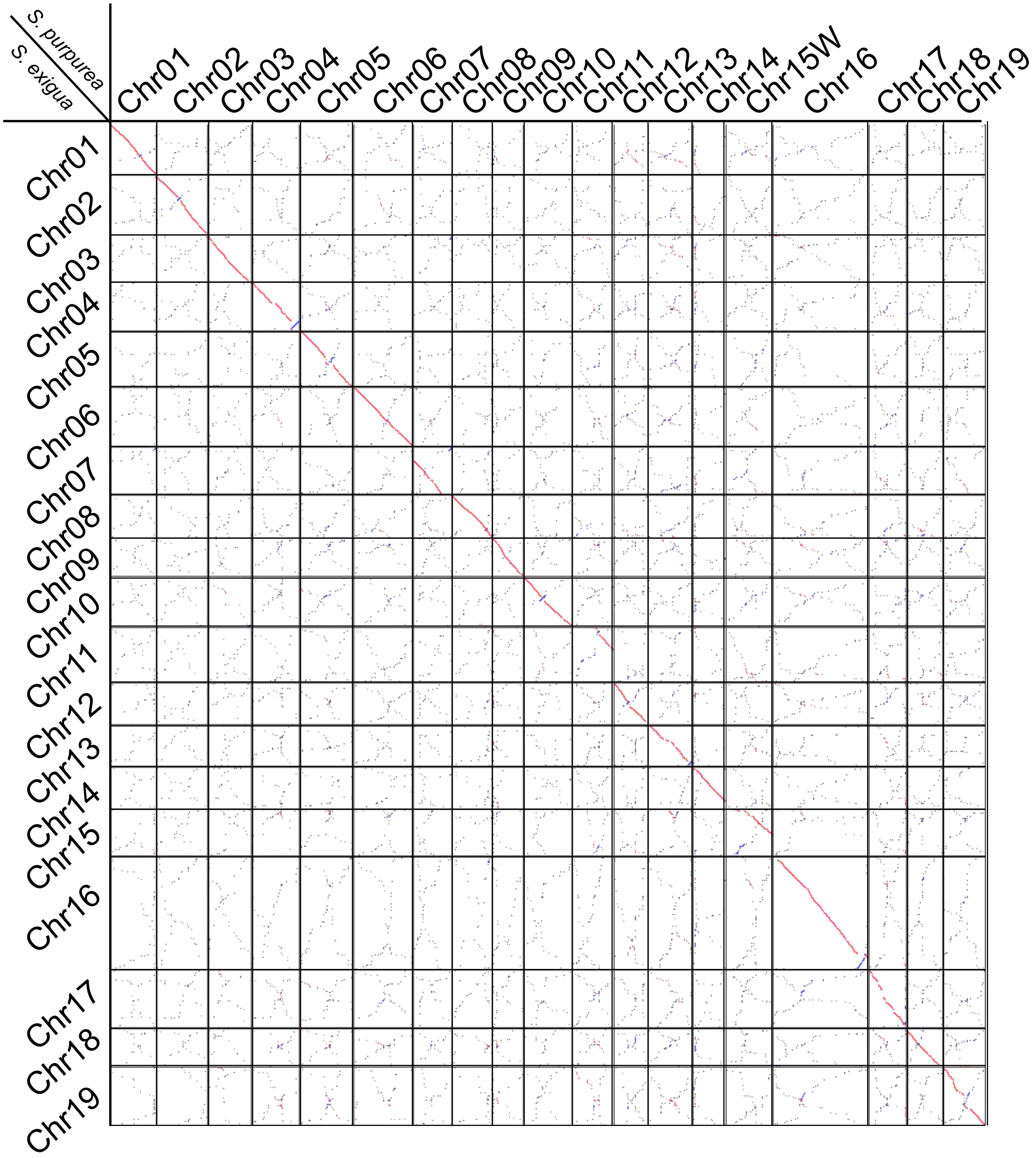

Figure S1. All-to-all alignment between our *S. exigua* male assembly and the *S. purpurea* reference genome v5.1 from JGI

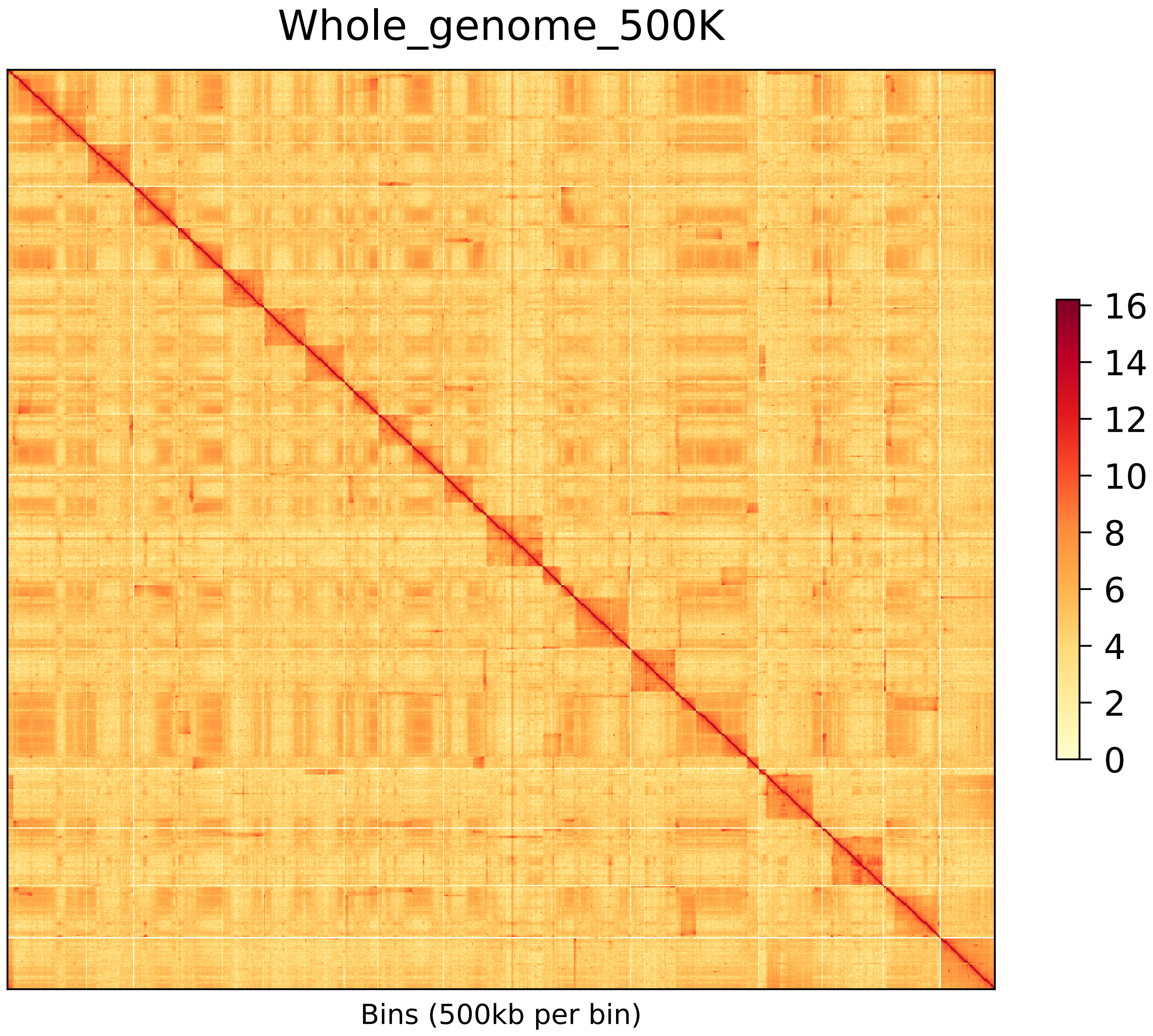

Figure S2. Hi-C interaction map of the *S. exigua* assembly generated by 3D-DNA (Dudchenko et al. 2017). Higher interactions (more red) indicate chromosome interaction signals between two segments related to close physical proximity between DNA segments. A Hi-C interaction block (which look like squares) represents a physical linkage group with low chromatin interactions with other groups.

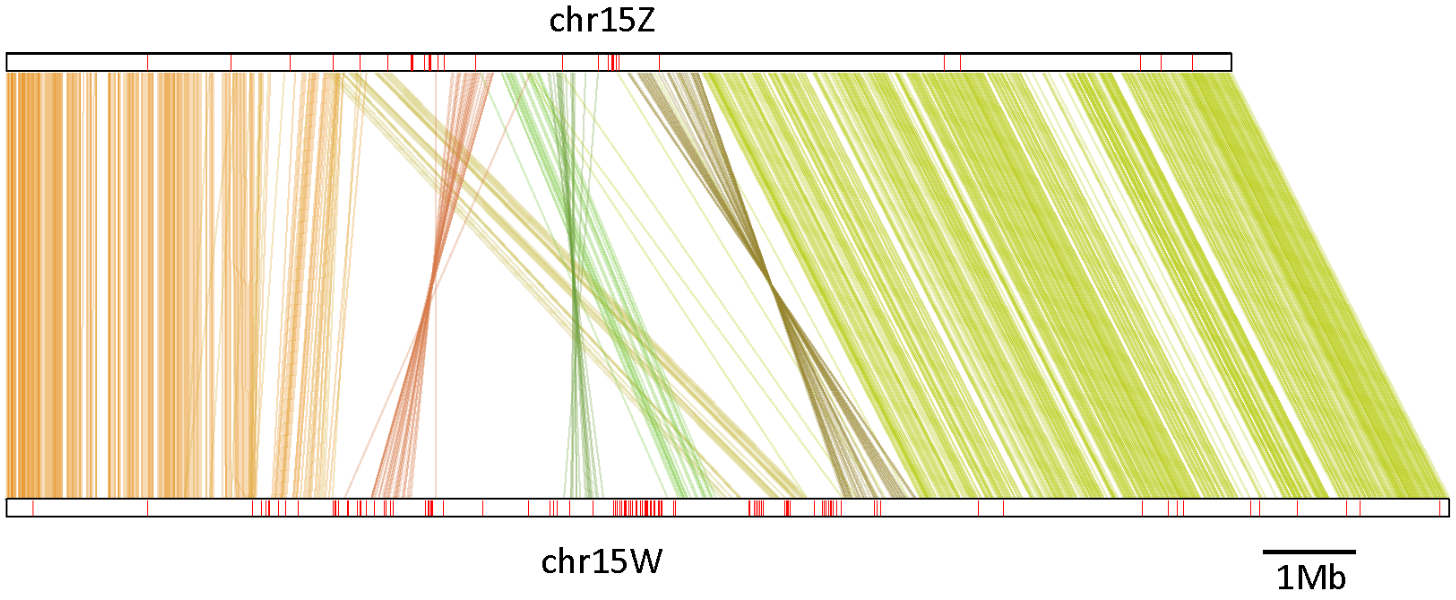

Figure S3. Synteny of Chr15Z and Chr15W. Colors between Chr15Z and Chr15W indicate synteny groupings. Red lines on each chromosome suggest chromosome-specific genes (genes that do not have blastp results on the other chromosome).

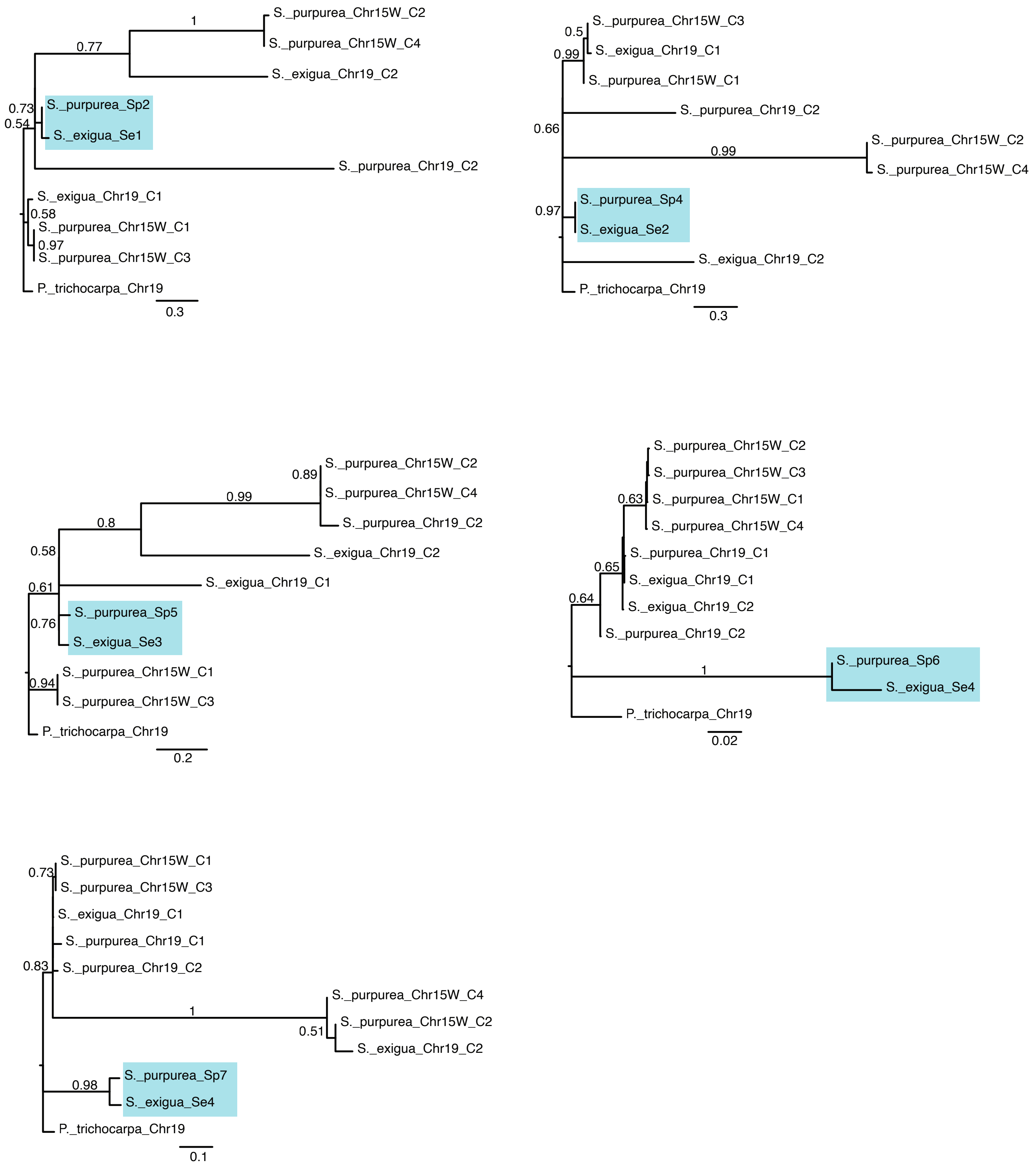

Figure S4. Homologous partial duplicates on the Y and Z chromosomes have a single origin. Phylogenies include homologous partial duplicates (highlighted in blue) on *S. exigua*, *S. purpurea* Z, and homologous regions on the intact copy of *RR17* in *S. exigua*, *S. purpurea*, and *P. trichocarpa*. Branch lengths indicate the numbers of nucleotide changes. The patterns suggest that the partial duplicates on Z and Y each have a single origin. Bootstrap support >70 is indicated. The tip labels are formatted as Species-Chromosome-Copy number. C refers to the intact copies of *RR17*.

Supplementary References

Dudchenko, O. *et al.* De novo assembly of the *Aedes aegypti* genome using Hi-C yields chromosome-length scaffolds. *Science* **356**, 92-95, doi:10.1126/science.aal3327 (2017).
