# Supplementary material for: Evolution of a ZW sex chromosome system in willows
Source: Nat Commun. 2023 Nov 6;14:7144. doi: 10.1038/s41467-023-42880-5 (PMC10628195; doi:10.1038/s41467-023-42880-5)
Supplement: Supplementary file 3 — Description of Additional Supplementary Files [file 41467_2023_42880_MOESM3_ESM.pdf]

Description of Additional Supplementary Files

Supplementary Data 1
Description: Sex-associated positions of S. exigua when mapping to S. exigua and S. purpurea reference genome using genome-wide association study.

Supplementary Data 2
Description: Contigs assembled to Chromosome 15 in S. exigua male (#SE967M) with Hi-C interacting map. "fragment_#" refers to a contig with a substantial portion of the Nanopore assembly

Supplementary Data 3
Description: Colinearity between Contig 511 of S. exigua and Chr15Z of S. purpurea.

Supplementary Data 4
Description: Shared ancestry among Y, Z, and W implied by similarities on sex-associated alleles.

Supplementary Data 5
Description: BLASTn output of P. trichocarpa RR17 sequence aligned to S. purpurea genome and S. exigua genome.

Supplementary Data 6
Description: Positions of RR17 partial duplicates between S. exigua and S. purpurea
