# Supplementary material for: Evolution of a ZW sex chromosome system in willows
Source: Nat Commun. 2023 Nov 6;14:7144. doi: 10.1038/s41467-023-42880-5 (PMC10628195; doi:10.1038/s41467-023-42880-5)
Supplement: Supplementary file 10 — Reporting Summary [file 41467_2023_42880_MOESM10_ESM.pdf]

nature portfolio

Corresponding author(s): Matthew Olson

Last updated by author(s): Oct 20, 2023

Reporting Summary

Nature Portfolio wishes to improve the reproducibility of the work that we publish. This form provides structure for consistency and transparency in reporting. For further information on Nature Portfolio policies, see our Editorial Policies and the Editorial Policy Checklist.

Statistics

For all statistical analyses, confirm that the following items are present in the figure legend, table legend, main text, or Methods section.

n/a	Confirmed	
☐	☒	The exact sample size (n) for each experimental group/condition, given as a discrete number and unit of measurement
☐	☒	A statement on whether measurements were taken from distinct samples or whether the same sample was measured repeatedly
☐	☒	The statistical test(s) used AND whether they are one- or two-sided<br>*Only common tests should be described solely by name; describe more complex techniques in the Methods section.*
☒	☐	A description of all covariates tested
☐	☒	A description of any assumptions or corrections, such as tests of normality and adjustment for multiple comparisons
☒	☐	A full description of the statistical parameters including central tendency (e.g. means) or other basic estimates (e.g. regression coefficient) AND variation (e.g. standard deviation) or associated estimates of uncertainty (e.g. confidence intervals)
☐	☒	For null hypothesis testing, the test statistic (e.g. F, t, r) with confidence intervals, effect sizes, degrees of freedom and P value noted<br>*Give P values as exact values whenever suitable.*
☒	☐	For Bayesian analysis, information on the choice of priors and Markov chain Monte Carlo settings
☐	☒	For hierarchical and complex designs, identification of the appropriate level for tests and full reporting of outcomes
☒	☐	Estimates of effect sizes (e.g. Cohen's d, Pearson's r), indicating how they were calculated

Our web collection on statistics for biologists contains articles on many of the points above.

Software and code

Policy information about availability of computer code

Data collection	no software was used for data collection
Data analysis	For genome assembly: Flye v2.9-b1178, Porechop v0.2.4, Guppy, Nextpolish v1.3.1, Juicer v.1.5, 3d-dna v180922. For alignment: lastz v1.1.13,BLASTN 2.6.0. For SNP calls: BWA-memv0.7.17-r1188, GATK 4.0.12.0. For GWAS: Plink v1.90b4. For alignments and phylogenies: CLUSTALW, MEGA11. For plotting: R 4.2.1, last-dotplot Build 1333

For manuscripts utilizing custom algorithms or software that are central to the research but not yet described in published literature, software must be made available to editors and reviewers. We strongly encourage code deposition in a community repository (e.g. GitHub). See the Nature Portfolio guidelines for submitting code & software for further information.

Data

Policy information about availability of data

All manuscripts must include a data availability statement. This statement should provide the following information, where applicable:

- Accession codes, unique identifiers, or web links for publicly available datasets
- A description of any restrictions on data availability
- For clinical datasets or third party data, please ensure that the statement adheres to our policy

The Oxford Nanopore (ONT) reads generated in this study are deposited in the NCBI BioProject under accession number PRJNA1010806 [https://

www.ncbi.nlm.nih.gov/bioproject/PRJNA1010806]. The Illumina and Hi-C interaction mapping reads generated in this study for genome assembly polishing are deposited in the NCBI BioProject under accession number PRJNA1010212 [https://www.ncbi.nlm.nih.gov/bioproject/PRJNA1010212]. The population sequence capture array Illumina reads generated in this study have been deposited in the NCBI BioProject under accession number PRJNA1009225 [https://www.ncbi.nlm.nih.gov/bioproject/PRJNA1009225]. The genome assembly of male and female Salix exigua have been deposited in the NCBI BioProject under accession number PRJNA1009227 [https://www.ncbi.nlm.nih.gov/bioproject/PRJNA1009227] and PRJNA1009230 [https://www.ncbi.nlm.nih.gov/bioproject/PRJNA1009230]

Research involving human participants, their data, or biological material

Policy information about studies with human participants or human data. See also policy information about sex, gender (identity/presentation), and sexual orientation and race, ethnicity and racism.

Reporting on sex and gender	n/a
Reporting on race, ethnicity, or other socially relevant groupings	n/a
Population characteristics	n/a
Recruitment	n/a
Ethics oversight	n/a

Note that full information on the approval of the study protocol must also be provided in the manuscript.

Field-specific reporting

Please select the one below that is the best fit for your research. If you are not sure, read the appropriate sections before making your selection.

☐ Life sciences ☐ Behavioural & social sciences ☒ Ecological, evolutionary & environmental sciences

For a reference copy of the document with all sections, see nature.com/documents/nr-reporting-summary-flat.pdf

Ecological, evolutionary & environmental sciences study design

All studies must disclose on these points even when the disclosure is negative.

Study description	We mapped sex determination in Salix exigua and analyzed the movement of sex chromosomes in the genus.
Research sample	We sampled 48 Salix exigua individuals - 24 males and 24 females - for mapping sex determination regions in the Rio Bonito population. This sample size has been shown sufficient for mapping SDRs in many studies. We chose to sample S. exigua because it represents a previously unsampled taxonomic section of Saix. The population sample is meant to represent sex chromosomes in the species,
Sampling strategy	Plants were marked in the field and sampled arbitrarily along a linear transect following the bank of the Rio Bonito. Arbitrary sampling was reasonable because we did not know the genotypes of individuals other than by knowing their sexes. To avoid sampling the same clone, we collected from trees that were separated by >10m or were obviously different individuals because one was a male and other other was a female. We sampled 24 males and 24 females. This sample size had been shown in previous studies (Sanderson et al. 2021 Heredity) and in independent studies (Muyle et al. 2016 GBE) to be sufficiently large for detecting sex determination regions with high power (0.8).
Data collection	Field data were recorded on the sexes of individuals in a field notebook by Nan Hu and Haley Hale.
Timing and spatial scale	Leaf samples from 48 individuals were collected for sequencing in early April 2017. All samples were collected along a ~2 km transect. Samples for genome assemblies were collected from the same population on October 20, 2018.
Data exclusions	no data were excluded
Reproducibility	The study question of identifying the sex chromosome does not require replicated studies. These GWAS studies are not usually reproduced.
Randomization	Genotypes were assumed to be randomly distributed among the collected samples. A posteriori analyses of genetic diversity supported this assumption.
Blinding	Not applicable. No studies with manipulated treatments that would require blinding ere preformed for this research.

Did the study involve field work? ☒ Yes ☐ No

Field work, collection and transport

Field conditions	We collected during one day. It was sunny.
Location	33º31'39.33"N 150º26'21.05"W on the Rio Bonito on Fort Stanton-Snowy River Cave National Conservation Area, Bureau of Land Management USA
Access & import/export	In 2017, Haley Hale obtained permission for sampling from Michael McGee at the the main office at the Fort Stanton-Snowy River Cave National Conservation Area, Bureau of Land Management (BLM) USA. No samples were imported or exported across national borders. The BLM land where leaf tissues were collected has no restrictions on plant collecting.
Disturbance	We did not disturb the area.

Reporting for specific materials, systems and methods

We require information from authors about some types of materials, experimental systems and methods used in many studies. Here, indicate whether each material, system or method listed is relevant to your study. If you are not sure if a list item applies to your research, read the appropriate section before selecting a response.

Materials & experimental systems

n/a	Involved in the study	
☒	☐	Antibodies
☒	☐	Eukaryotic cell lines
☒	☐	Palaeontology and archaeology
☒	☐	Animals and other organisms
☒	☐	Clinical data
☒	☐	Dual use research of concern
☐	☒	Plants

Methods

n/a	Involved in the study	
☒	☐	ChIP-seq
☒	☐	Flow cytometry
☒	☐	MRI-based neuroimaging

Dual use research of concern

Policy information about dual use research of concern

Hazards

Could the accidental, deliberate or reckless misuse of agents or technologies generated in the work, or the application of information presented in the manuscript, pose a threat to:

No	Yes	
☒	☐	Public health
☒	☐	National security
☒	☐	Crops and/or livestock
☒	☐	Ecosystems
☒	☐	Any other significant area

Experiments of concern

Does the work involve any of these experiments of concern:

No	Yes	
☒	☐	Demonstrate how to render a vaccine ineffective
☒	☐	Confer resistance to therapeutically useful antibiotics or antiviral agents
☒	☐	Enhance the virulence of a pathogen or render a nonpathogen virulent
☒	☐	Increase transmissibility of a pathogen
☒	☐	Alter the host range of a pathogen
☒	☐	Enable evasion of diagnostic/detection modalities
☒	☐	Enable the weaponization of a biological agent or toxin
☒	☐	Any other potentially harmful combination of experiments and agents
